# Conspiracy theories on Twitter: emerging motifs and temporal dynamics during the COVID-19 pandemic

**DOI:** 10.1007/s41060-021-00298-6

**Published:** 2021-12-24

**Authors:** Veronika Batzdorfer, Holger Steinmetz, Marco Biella, Meysam Alizadeh

**Affiliations:** 1 Computational Social Science, GESIS – Leibniz Institute for the Social Sciences, Cologne, Germany; 2 Faculty of Management, University of Trier, Trier, Germany; 3 Department of Psychology, Eberhard Karls Universität Tuebingen, Tuebingen, Germany; 4 Kennedy School of Government, Harvard University, Cambridge, USA

**Keywords:** Word embedding, COVID-19, Time series analysis, Conspiracy beliefs, Twitter structural break analysis

## Abstract

The COVID-19 pandemic resulted in an upsurge in the spread of diverse conspiracy theories (CTs) with real-life impact. However, the dynamics of user engagement remain under-researched. In the present study, we leverage Twitter data across 11 months in 2020 from the timelines of 109 CT posters and a comparison group (non-CT group) of equal size. Within this approach, we used word embeddings to distinguish non-CT content from CT-related content as well as analysed which element of CT content emerged in the pandemic. Subsequently, we applied time series analyses on the aggregate and individual level to investigate whether there is a difference between CT posters and non-CT posters in non-CT tweets as well as the temporal dynamics of CT tweets. In this regard, we provide a description of the aggregate and individual series, conducted a STL decomposition in trends, seasons, and errors, as well as an autocorrelation analysis, and applied generalised additive mixed models to analyse nonlinear trends and their differences across users. The narrative motifs, characterised by word embeddings, address pandemic-specific motifs alongside broader motifs and can be related to several psychological needs (epistemic, existential, or social). Overall, the comparison of the CT group and non-CT group showed a substantially higher level of overall COVID-19-related tweets in the non-CT group and higher level of random fluctuations. Focussing on conspiracy tweets, we found a slight positive trend but, more importantly, an increase in users in 2020. Moreover, the aggregate series of CT content revealed two breaks in 2020 and a significant albeit weak positive trend since June. On the individual level, the series showed strong differences in temporal dynamics and a high degree of randomness and day-specific sensitivity. The results stress the importance of Twitter as a means of communication during the pandemic and illustrate that these beliefs travel very fast and are quickly endorsed.

**Supplementary Information:**

The online version contains supplementary material available at 10.1007/s41060-021-00298-6.

## Introduction

Humans are prone to search for causal explanations of events driven by the need to learn and adapt. Among the myriad of event types, the interpretation of social and political events is especially important as these may lead to exploitation or other threats for the individual or group. As an extreme form of interpreting events, conspiracy theories (CTs), that is, sets of beliefs about the existence of a hidden and powerful coalition of people or organisations with malevolent agendas, have become a prominent research field [1]. This is partially due to the assumption that CTs may prompt a radicalisation process in which individuals develop beliefs immune to falsification [2]. Additionally, as CTs often trigger the need to defend against perceived threats, they may elicit behaviour either detrimental to the individual (e.g. isolation) or the social environment (e.g. deviant behaviour).

Research shows that crisis situations and dramatic events (e.g. natural disasters) or terror events cause a high level of uncertainty and, thus, foster the emergence of conspiracy ideation [3, 4]. Such events are usually complex, while their causes and remedies are unknown, as media coverage is most often contradictory and incomplete. In order to rationalise such phenomena and decrease personal uncertainty and lack of control, rumours and conspiratorial ideation might provide coping strategies for collective sensemaking. One instantiation of such a situation is the COVID-19 pandemic that started at the beginning of 2020. Not only has the uncertainty about the spread of the disease affected collectives and individuals but also the resultant public health interventions (e.g. the range of non-pharmaceutical measures being implemented by governments around the globe with a direct impact on social, economic lives and individual behaviours and well-being) [5]. These public health interventions profoundly impacted sensemaking (e.g. distrust of authorities) and behavioural responses (e.g. decreased willingness for vaccination or increase in deviant behaviour) [6–8].

In recent years, social media platforms have not only become a viable means for individuals to inform themselves but also a platform to disseminate conspiracy ideation [9]. As such, these platforms are not only the relevant environment where CTs evolve but also a viable data source for research. This latter aspect of platforms leads us to the question how to gather text that is indicative of CTs beyond the simple focus on predetermined search terms, which make compiling an exhaustive list of synonyms and related concepts a challenging task. More importantly, in contrast to past research utilising keywords or hashtag-based identified samples of CT users, focusing on derogatory language or taking keywords alone as a sufficient indicator for a CT user, we adopt an iterative procedure that has a theoretical foundation in evolutionary psychology (i.e. not every remark about Bill Gates represents a CT). As a remedy, with the approach from distributional semantics we are able to delineate tweets which co-occur with each other and hence hold related semantic meaning and which serve to expand our initial scope. Most notably, we do not presume that every posting by a CT user is in fact a conspiracy tweet. This allows for differentiated individual human behaviour as degrees of engagement with a concept that is derived from theory, alongside the variability of postings over time.

Likewise, the focus on social media platforms allows an in vitro view on the temporal dynamics of content creation and communication by means of an intensive longitudinal perspective. As any other behaviour, expressing CTs is a temporal process with probable nonlinear dynamics involving slow trend changes as well as abrupt chaotic spikes. Investigating these dynamics can provide insights on the psychological underpinnings and their rational and strategic versus affective and impulsive characteristics. Likewise, phenomena such as inertia and long-term trend changes can give insights into possible radicalisation processes in which people, when considering CTs, create a positive feedback loop, resulting in respective behaviour for a period of time or even in a durable fashion.

The present paper aims at exploiting these two merits of social media platforms. First, by using word embeddings, we investigate CTs utilising a data-based approach (i.e. *vector semantics*) that assigns meaning to a word by the distribution of words around it, combined with paradigmatic examples. With this natural language processing approach, we explore the context around COVID-19 discourse from a semantic perspective, in a time span when the conspiracy beliefs and narratives have emerged and spread. Second, to analyse the temporal dynamics, we apply a time series perspective [10] and investigate, in an unobtrusive way, the temporal characteristics of user behaviour on social media as collective responses alongside individual ones. In this regard, we provide a description of the series of tweets both aggregated across individuals and individual series, conducted an STL decomposition in trends, seasons and errors, as well as an autocorrelation analysis [11]. We further attend to applied generalised additive mixed models to analyse nonlinear trends and their differences across users [12]. Moreover, we conducted a structural break analysis of the series of CT tweets in 2020 that could provide hints on the responsiveness to external events [13].

In particular, the paper adopts an exploratory perspective and aims to answer the following questions: (1) Which CT motifs emerged in the pandemic, and which terms are indicative of these motifs? (2) Do the CT group and non-CT group differ in the temporal dynamics of their posting behaviour of overall COVID-19-related content—that is, are there differences in the nonlinear trends, within-week rhythms of posting (i.e. *seasonality*), and degree of autocorrelation indicating inertia vs. randomness of tweets? (3) What are the temporal dynamics (e.g. trends, seasonality, autocorrelation) of CT tweets, and (4) are there inter-individual differences between users in these dynamics? The difference between processes on an aggregate versus individual level is a dominant issue in the social sciences. In this regard, scholars have repeatedly stressed not to trivially generalise results from one level to the other, both, with regard to social systems in general [14], and culture or social media, in particular [15].

To answer these questions, we present results of a social media analysis of *N* = 218 Twitter users (among them *n* = 109 CT posters) who have tweeted content with CT content over a period of approx. 11 months (from January until November 2020). This group is contrasted with *n* = 109 Twitter users who have not posted messages containing CTs (i.e. non-CT posters). Our study offers two major contributions, that is, firstly, providing a proof-of-concept to differentiate conspiracy language and to characterise it by linguistic similar indicators and psychological needs. Secondly, we assess how time series methods can enrich a theory-rooted view on dynamic user engagement. More specifically, we deliver an important contribution to the data science community, which rests on a substantive theoretical basis on which we build our automated NLP pipeline and time series analyses. The theoretical concepts—in our case these are concepts stemming from evolutionary psychology—aim to characterise forms of individual engagement with conspiracy content (regarding content types, as well as differentiating CT opinions from non-CT content). We deem such a theoretical foundation as fruitful for three reasons: First, distinguishing the variability of individuals in voicing conspiracy content and some of the underlying motivations against aggregated system dynamics allows us to analyse individual behaviour in a social context. Second, considering our temporal focus, we gain knowledge about trends (and their variability across users) that provide information about a possible radicalisation as well as temporal characteristics of the posting behaviour (i.e. whether it is systematic vs. impulsive) or structural breaks (as system responses to shocks that may hint at coping behaviour or persistent maladaptations) which can be taken into consideration when developing interventions [11]. Third, differentiating CT content from non-CT content with an approach from distributional semantics is scalable. In the next section, we provide the theoretical background on conspiracy theories that provides the basis of our word embedding approach.

## Background

### Conspiracy theories (CTs)

A plethora of definitions regarding conspiracy theories exist that are at times contradictory and reflect a phenomenon that is hard to actionably delineate [16]. Likewise, as understanding the minimal sufficient determinants for radicalisation processes, frameworks span pathological manifestations, cognitive or trait explanations, yet few approaches adopt an actionable definition [17]. We depart from a view on CTs that are defined as the belief that hidden coalitions of powerful individuals follow an agenda that intends or causes harm to society, the particular in-group of the individual, or the individual specifically. While mistrust, criticisms, and specific claims are often erroneously regarded as CTs, van Prooijen and van Vugt [2] pointed out five criteria that define a CT which are adopted for this study. The first criterion is the perception of a *pattern* that leads individuals to connect events or specific observations to an integrated whole. Second, individuals assume an underlying *agency*, that is, they attribute intentionality of actions. This propensity results from the overall tendency to form social knowledge that strives to understand and predict human decisions and their behaviour. Third, people assume the joint acting of *coalitions–*in the vast majority of a more powerful group compared to one’s own group. Fourth, the person thinks the plans of this group present a *threat* to the person or in-group, and fifth, either the group or its plans are *secret*, which makes it difficult to find clear evidence for the convictions and falsify them.

While research and especially the public discussion tend to view CTs as irrational, an expression of a pathological mind [18], or an extremist political attitude, van Prooijen and van Vugt [2] emphasise the evolutionary roots of CTs as a functional adaptation to persisting actual threats by hidden coalitions or at least side-products of specific functional adaptations, such as the tendency for pattern recognition or harm detection sensitivity. They note, however, that while being functional for the vast history of humans, this “hyperactive agency-detection system” (p. 773) has lost its usefulness in modern society, and CTs are now the result of this innate sensitivity being confronted by apparent cues, ubiquitous in the internet and social media era.

Adopting a more psychological perspective, Douglas et al. [1] claim that CTs serve the fulfilment of three basic needs–an *epistemic need* to understand the world and the causes and consequences of relevant events, an *existential need* to avoid harm, achieve security, control the environment, and a *social need* to preserve a positive social identity. In particular the latter helps to understand that CTs often evolve, caused by the perception of intergroup conflicts, discrimination, or relative deprivation (e.g. [19, 20]). Douglas et al. [1] stress that although CTs aim to fulfil these needs, they fail to do so. Specifically, epistemic needs are unfulfilled as the individual creates CTs immune to falsification, unrealistically complex, irrational, and unfounded. Likewise, the need to gain control and reduce uncertainty is unmet as the individual increases his/her perception of being the victim of powerful others. Empirically this has been shown to lead to reduced activities that actually would increase control (e.g. political engagement [21]). Likewise, CTs result in ongoing resentment and distrust against other groups or institutions that, in the long term, excel immediate feelings of superiority of the in-group.

Empirically, research on CTs has evolved in a variety of fields, such as psychology, political science, sociology, medicine, or anthropology. Topics have been likewise diverse, ranging from overall theoretical discussions [22, 23], anti-science CTs, often discussed with the example of anti-climate change CTs [24–26] or anti-vaccination CTs [27–29], and the role of demographic predictors [20, 30], political predictors, such as political orientation [31, 32], or psychological predictors [33–36].

An additional reason why investigating conspiracy beliefs is crucial in the COVID-19 context is related to the link between these beliefs and the rejection of scientific knowledge. Specifically, conspiracist ideation has been linked to greater opposition to scientific advancements such as vaccinations and climate science [26]. Moreover, conspiracy content has been found on many online platforms [37]. These types of content allude to for-profit collusion between vaccination promoters and pharmaceutical companies or cover-ups hiding the vaccine's side effects, while it promotes "rebel doctors" who break away from medical establishments, refusing to support scientifically supported policies. Moreover, they relate conspiracy theories to the COVID-19 situation as they can be easily spread over social media, such as Twitter, contributing to the dangerous impact of these media on vaccination hesitancy [38]. Given that time series approaches are scarce in the literature on conspiracy theories, we introduce central concepts of times series analyses in the following section.

### Understanding the dynamics of CTs

Behaviour on social media is of scientific and practical interest because of the low barriers to post content and, thus, the high chances to be able to analyse impulsive actions due to emotional processes or reactions to stimuli (e.g. news events). Such social media communication may affect public risk perceptions during other crisis events, like the Zika virus in the USA in 2016 [39]. Research found that CTs identified in social media posts differed from rumours in their temporal pattern, that is, CTs peaked multiple times in a period, whereas rumours showed single peaks and recession patterns [40]. Further, the long-term elaboration and reinvention of CTs was shown in work by Nied et al. [41], indicating that respective groups on Twitter comprise individuals with diverse ideologies and beliefs. This sets the stage for fruitfully investigating the particularities vs. generality of online posting behaviour across time.

While fields such as economy or ecology have a tradition in investigating dynamic processes, only recently the behavioural sciences adopted such approaches. Among these, time series analysis has been applied considerably seldom [42]. This is disadvantageous, as beyond their methodological capabilities, time series concepts have theoretical benefits due to the possibilities for conceptualising temporal dynamics. This is the case for the occurrence of *linear* or *nonlinear trends*, *autocorrelation* [43], *systematic fluctuations* (periodicity and seasonality, see [44]), and *structural breaks* in mean level, trend, or variance [45] that show the behaviour of the system in response to sudden and emerging external or internal events. These concepts all share the fundamental purpose that we learn something about the underlying dynamics of psychological entities (e.g. beliefs and attitudes), emotional processes and their rhythms, regulations (or their failure), and long-term (mis)adaptations versus learning in the form of ongoing disequilibria.

### Aggregate versus individual dynamics

With regard to the analyses of the temporal characteristics of the posting behaviour, our paper considers these characteristics on the aggregate level (i.e. the sum of postings of the overall groups) as well as on the individual level that focuses on individual users and their differences. By doing so, our approach adopts a multilevel perspective that is ubiquitous in the social sciences. Research involving hierarchical systems conceives individual entities (e.g. individuals) nested within higher-order entities (e.g. work teams, organisations, or other collectives). Inherent in this perspective is the emphasis that characteristics of the various levels are ontologically different, with the most prominent concept being “emergent systems” stressing that higher order entities may have a unique ontological status that cannot be deduced by its components. In the case of posting behaviour, an aggregate perspective, inspecting a part of the collective may be fruitful as an instantiation of collective sensemaking. Beyond these ontological issues, scientific approaches across disciplines have always been subject to the difference between *nomothetic* versus *idiosyncratic* perspectives and potential generalisations of scientific results versus particularities. In the most extreme example, single case designs have emerged but remained limited in their popularity [46]. Other scholars, in contrast, have proposed that both perspectives should not be viewed as contradictions; rather, studies investigating both perspectives should be conducted. In our paper we follow the latter perspective and analyse the aggregate series in addition to the individual series and their differences.

## Research questions

As aforementioned, our study intends to apply word embeddings to identify terms signifying CTs and their semantic relationships and to analyse how CT tweets unfold over the first year of COVID-19 in 2020. By these means, we learn the temporal characteristics of tweeting CTs, their trends, dynamic profile, extent of external sensitivity, and systematic versus impulsive (or random) parts. To this end, we compare different groups of people and series of different content (CTs vs. overall COVID-19-related content). We emphasise that the comparison does not aim to explain differences between the groups beyond the characteristics of their posting behaviour.

In particular, we investigate the dynamics of CTs from two perspectives. Namely, we differentiate the aggregate level and the individual level and potential differences in their ontological status, processes, and temporal dynamics. Table 1 summarises the research questions.

**Table 1:** Levels of research questions

	CT group	Non-CT group
Aggregate level (averaged tweets)	**RQ1:** Identify semantically similar expressions of CTs	
	**RQ2**: Comparison of the posting behaviour on overall coronavirus-related (i.e. non-CT) content	
	**RQ3**: Identify temporal characteristics of CT tweets	
Individual level	**RQ4**: Individual differences in temporal characteristics	

*CT* Conspiracy theory, *RQ* Research question

On the aggregate level, we focussed on the temporal characteristics of the overall posting behaviour and investigated the total proportion of tweets posted across each day between January and November of 2020. The *CT group* consists of individuals who posted conspiracy-related content. To identify differences versus commonalities with Twitter users who do not post CTs and their posting behaviour, we identified a *non-CT group*. The first research question (RQ1) relates to characterising CT tweets from the CT group not only in terms of what information they post but also how users formulate their posts of coronavirus-related content. For this purpose, we use word embeddings to identify semantically similar term vectors underlying the concepts.

The second research question (RQ2) focussed on the comparison of the two groups. To have a common ground, we directed our attention towards tweets with COVID-19-related content, containing information on infection rates, social distancing measures, recommendations to wear a mask, etc. We explicitly excluded CT tweets (see Methods section on word embeddings).

With the third research question (RQ3), we analysed the temporal characteristics of the CT-related tweets. To this end, we focussed on two relevant variables: The overall number of active users posting CTs each day and the mean proportion of CT tweets of all tweets across the days. Of particular interest was the analysis of a potential trend in the number of users and proportion of tweets. Further, the autocorrelations examined for the proportion of CT tweets provide information on potential inertia and, thus, the degree of recovery of the aggregate to mean levels. Finally, the exploration of structural breaks can give rise to interpretations of external events causing these changes.

Finally, the fourth research question (RQ4) focussed on the individual level of analysis. Here, we aim at analysing the series of tweets for each user separately. Consequently, estimation of their trends across 2020 allows us to learn about differences between individuals—most notably differences in the functional form of trends (i.e. linear vs. nonlinear) and directions (i.e. upward trends vs. downward trends). In addition, differences in the autocorrelation coefficients indicate differences in inertia vs. fast recovery (e.g. after emotionally triggering news). In summary, the inter-individual approach provides an empirical basis for future research targeting predictors or explanatory factors of these differences.

## Method

### Data collection and preprocessing

#### Information retrieval

In order to identify CT users, we manually searched for matching keywords in the advanced search of the web version of Twitter and then retrieved the matching tweets and tweet handles. This offers the opportunity to model individual trajectories over time, capture the occurrence of the target keywords at different points in the year, and not be compromised by algorithmic sampling biases that favour the most recent, trending postings. We collected a list of thematic keywords, potentially indicative for conspiracy theories, based on research articles [47, 48], and third-party sources [49]. The selection was based primarily on the occurrence of thematic topics that were flagged as misinformation by the “EUvsDISNFO”-database in 2020 [50]. For more details on the sources, coding scheme, and examples please see the Supplementary Materials 1.1–1.3). The search queries comprised the bespoken keywords, as well as a reference to the broader COVID-19 context (e.g. ‘pandemic’). Using each of these keywords, we queried the Twitter user interface for a seven-month period (see Supplementary Materials 1.2) and retrieved users with matching tweets for each month. By sampling per month 10 random users that matched the queries resulting in 420 sampled and annotated tweets, we are able to capture users over the year of 2020, in contrast to sampling with the Streaming API or Search API, which are primarily used for forward searches. Thereby, we could address the potential bias of only sampling users that have been highly active in a recent short time interval of the particular sampling time. Subsequently, we employed two independent coders who annotated user’s tweets (as containing potential CT content). For the annotation, only original tweet content was considered, to avoid confounding by third-party opinions (e.g. retweets). We retained, however, links to external sources or sharing of picture material. The underlying coding scheme was based on a catalogue of five criteria (comprising: agency, secrecy, coalition, threat, pattern) of which at least three criteria needed to be met in a tweet to be indicative for expressing a CT, along biographical information. Cohen’s kappa was assessed on the binary decision of including or excluding an account. In particular, coders were trained on Twitter-specific platform affordances (posting types and conventions, non-standard abbreviations and symbols) and conspiracy-specific characteristics (e.g. examples for deceptive intentions or coalitions). A sample of 10 random users was coded as a pre-test. Subsequently, we turned to the whole dataset of 420 matching tweets and a first round of coding was conducted. We achieved a Cohen’s Kappa of *κ* = 0.60, which indicates an agreement of 79.76% [51]. After the initial coding, in a second round, disagreement between raters on ironic or allusive tweets (e.g. “swamp” referring to the deep state coalition) was resolved by consulting the respective tweet history and profile. This process of resolving disagreement led to the inclusion of *N* = 203 Twitter accounts. We acknowledge that potential conspiracy accounts might have been excluded due to factors such as extremely short tweets, incomprehensible abbreviations, lack of sentence structure, and the use of hashtags only.

To establish a non-CT group, we sought to identify Twitter users exhibiting a tweet behaviour focusing on coronavirus-related content but non-CT-related. For this purpose, we conducted a keyword search with Twitter’s Search API (e.g. corona OR covid OR pandemic) to identify common users.

#### Querying Twitter

In the next step, we used the Twitter REST API to harvest the available timelines (e.g. all tweets, retweets, or replies) of the selected accounts of both groups. This process referred to the public user timelines (i.e. the tweet history of the user) of each identified account and was conducted on 8 November 2020. For each of the timelines we were able to retrieve a maximum of 3,200 tweets, resulting in individual time series of unequal lengths. The unequal lengths imposed no limitations for the aggregate-level analyses, as we calculated the average percentage of tweets among all tweets.

#### User preprocessing

After retrieving the timelines, we applied the following criteria for inclusion of potential CT accounts as well as for the non-CT accounts (for the complete sampling flow, see Supplementary Materials 1.5–1.6). First, to remove dormant or entirely inactive accounts, we included only accounts that had posted status updates across a three-month period. Second, the accounts had to be owned by English-speaking users. In particular, we excluded accounts using English words at a rate of less than 80%, computed at the tweet level. Third, we used the R-package *tweetbotornot* [52] to exclude, with a probability of 80% and higher, accounts that were created by a bot. The functionality of the package takes into account features on the user level (e.g. profile information, account creation date) and tweet level (rate of status updates, or word complexity) [52]. In order to assess the extent of bias when classifying bots (false negatives or false positives), we manually annotated a random sample of initial CT accounts (40 out of 132) as well as non-CT accounts (40 out of 520). We based the classification on the user profile, the degree of human creativity and specificity of content, follower and friend count, extent of duplicates, and degree of automation (see [53]). We calculated the intercoder-reliability [54] for the CT accounts (*κ* = 0.6) and non-CT accounts (*κ* = 0.5). More false positives were found for the latter type, that is, human Twitter users were classified as a bot by TweetBotorNot. Eventually, after the filtering, this resulted in 109 accounts for the CT group and 333 accounts for the non-CT group, for the latter we drew a random sub-sample of 109 accounts. Pertaining to the face validity of the non-CT group, we drew a random sample of non-CT accounts (*n* = 40) from 109 accounts and annotated a random tweet, each of them by two coders, following the five-criteria scheme. Similarly, as with the CT group we calculated agreement for the binary categorisation of the tweet as conspiracy or not. This resulted in an agreement rate of 97.5 percent, with one tweet being flagged as conspiratorial.

#### Tweet preprocessing

We applied three steps of text pre-processing. First, tweets were converted to lowercase type, and then all links, HTML tags, ampersands, mentions, hashtag symbols, stopwords, and non-ASCII characters were converted or removed. Second, we converted expressions of emphasis from tweets (e.g. elongations such as ‘heyyyy’) to normal text. Third, we then tokenised the text using sets of up to two terms (i.e. two-grams). This procedure resulted in *N* = 142,559 tweets with 2,963,424 tokens for the CT posters as well as *N* = 95,394 tweets with 2,558,504 tokens for the non-CT posters.

### Distributional semantic model

Text documents from social media pose a substantive challenge when inferring latent information such as that which is needed for discriminating between conspiracy and non-conspiracy content in RQ1. Word embeddings, which range under the family of distributional semantic models, offer a state-of-the art approach for representing words in vector space to understand, at a word level, semantic meaning, but also to extract document similarity from them (here: tweets). More specifically, terms are represented with real numbers as a vector in continuous n-dimensional vector space, and the distance between the vectors denotes semantic similarity of the underlying construct. Word vectors exploit a spatial analogy, so that similar words have similar spatial relationships [55].

#### Global vectors for word representation

We use word embeddings for RQ1 to characterise emerging motifs with the respective related terms. More specifically, *Global Vectors for Word Representation* (GloVe) algorithm was used to discover latent vector representations in unannotated textual data [56]. With this method, word embeddings can be inferred from word co-occurrence matrices. GloVe is an unsupervised method for learning word representations based on log-bilinear regression that captures both global and local statistics of the term co-occurrence information [56]. This method of incorporating global statistics of word co-occurrences performs well even with small corpora [56]. Pennington and colleagues [56] showed, in experiments regarding the word analogy task, that a 100-dimensional GloVe model outperforms HPCA vectors or vLBL. The authors of GloVe argue for their approach by setting out that both count-based matrix factorisation methods and predictive neural network methods suffer several disadvantages [56]. Methods regarding global matrix factorisation consider statistical information, but they perform less optimally on internal evaluation tasks like the word analogy task that try to find semantically similar words [56]. Neural network methods like the *skip-gram* architecture (which try to predict the context word from a target word) perform better on the analogy task, but conversely show shortcomings on the global statistics of the corpus [56]. GloVe combines the best of both worlds as it allows us to consider the global context by the ratio of conditional probabilities to model the vector representations, as well as linear structures of vector spaces as likewise captured by *word2vec* [56].

Specifically, the GloVe algorithm uses co-occurrence probability ratios in the training phase of the word embeddings and accounts for rare co-occurrence word pairs [56]. The weighted least-squares objective function $J$ indirectly factorises the term–co-occurrence matrix $\left( X \right)$, where $w_{i} , w_{j}$ are word vectors and $V$ denotes vocabulary size [56] (see Eq. 1). The objective of the GloVe training is to minimise the difference between the dot product of word and context word vectors ($W_{i}^{T} \tilde{w}_{j}$) and the logarithm of the word co-occurrence probability of the word embeddings ($\log \left( {X_{ij} } \right)$). In order to avoid that rare co-occurrences are overweighted, a cost/weighting function $f\left( {X_{ij} } \right)$ is applied to the model (see Eq. 1). This function reduces the weight of co-occurrences appearing fewer times then the cut-off value *x*_max_.

1 $$ J = \sum\limits_{i,j = 1}^{V} {f\left( {X_{ij} } \right)\left( {W_{i}^{T} \tilde{w}_{j} + b_{i} + \tilde{b}_{j} - \log X_{ij} } \right)^{2} } $$

**Fig. 1:**
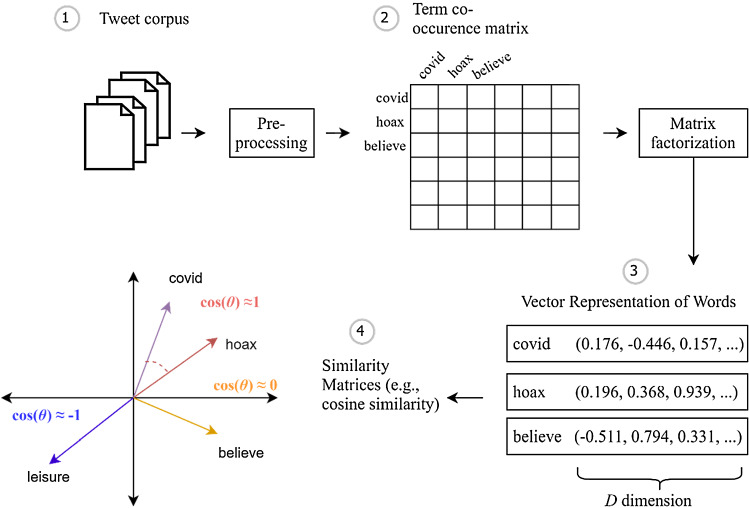
Framework for constructing *Global Vectors for Word Representation* (GloVe) models and measuring similarity

Our workflow for representing the respective tweet corpora as word embeddings (for each the CT group and the non-CT group) is shown in Fig. 1. Firstly, after pre-processing the raw tweets (step 1), we build a vocabulary of tokens (bigrams) from the corpus. These can then be represented as a global term-co-occurrence matrix (TCM) (step 2)—which takes into account the ratio of co-occurrence probabilities (by a pairwise context window). With GloVe, the cost function is directly optimised which allows for a more global context, as the dot product of two word vectors equals the number of times the terms co-occur. For the training, the GloVe algorithm uses the stochastic gradient descent algorithm to factorise the log of the TCM [57]. The resultant GloVe weight matrix consists of 2 vector types: main vectors and context vectors which are summed up [57]. Eventually, each token is represented as one real-valued vector of D-dimensions (step 3). The embedding dimensions in turn specify the complexity of the model and space into which we try to “embed” the tokens. The semantic similarity between two vectors can then be queried by similarity measures like cosine similarity (step 4).

Within this framework we vectorise text by (i) constructing symmetric, window-based TCMs from the pre-processed tweet “documents”, and (ii) fitting GloVe models to the TCM for CT posters and non-CT posters. In order to assess the GloVe model performance, we perform intrinsic evaluation (i.e. a word analogy task). This is a direct evaluation of the GloVe model performance—based on the hit-miss ratio of predicting a set of query terms and semantically related target words [58]. Here we used the BATS [59] and Google Analogy data set [60]. In our experimental setup we tested different settings for the hyperparameters. We tested the accuracy for different GloVe dimensions (50, 100, 150, 200, 250) and window sizes (3–12). As for the window size this denotes the context of a word that extends before and after a target term. Words that appear further away in the context from a word are given less weight than words closer to the respective word [56]. Thereafter, we adopted GloVe models with 100 dimensions and a context window of 8, which are simplest and showed best performance. We fixed the number of iterations to 20 and a convergence threshold of 0.001, so that training stopped if the maximum number of iterations is reached or the change in loss is lower than the convergence threshold. Furthermore, the number of co-occurrences within the weighting function $f\left( {X_{ij} } \right)$ denoted by *x*_max_ was set to 10.

#### Concept mover’s distance

In a next step tweets are discriminated against as conspiracy and non-conspiracy, ignoring user-related variables. This categorisation is guided by the semantic similarity of the word vectors with a custom CT lexicon, as well as a custom coronavirus lexicon. The general COVID-19 dictionary is based on the Yale Medicine vocabulary; hence, it comprises overall categories that relate to: linguistic variation of coronaviruses (e.g. “SARS”), medical-response (e.g. “remdesivir”), prevention (e.g. “stay_home”), spread of the disease (e.g. “outbreak”), and transmission (e.g. “symptomatic”) [61]. The CT dictionary comprised a set of seed terms as identified in the original article by van Prooijen and van Vugt [2], as well as by the EUvsDISNFO-database [50], as well as Part-of-Speech-Tagging of tweets (e.g. noun phrases for coalitions) for each category: agency (e.g. “plandemic”), threat (e.g. “eugenics”), coalition (e.g. “capitalist”), pattern (e.g. “great_awakening”), and secrecy (e.g. “mole”). Hence, the selection of seed terms connects to the initial five-category system of manual annotation, by building on these premises. The initial vocabularies for both dictionaries were enhanced by retrieving the 20 semantically most similar terms by cosine similarity of the GloVe vectors, associated with these seed terms, which were then selected based on relevance by human judgement.

**Fig. 2:**
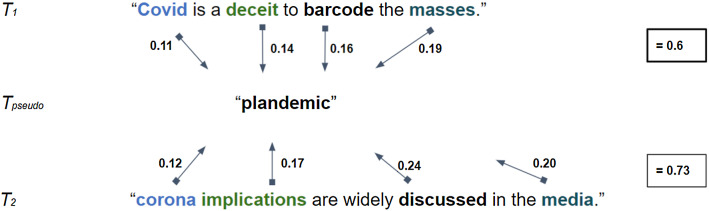
Illustration of the *Concept Mover’s Distance* principle (with $T_{1}$ and $T_{2}$ representing fictitious tweets and $T_{{{\text{pseudo}}}}$ a “pseudo”-document comprising only one term) (see also Kusner et al. [63])

Calculating the semantic similarity is achieved with the *concept mover’s distance* (CMD) algorithm [62]. As a development from the original *word mover’s distance* function [63], the CMD algorithm captures the semantic similarity between documents (i.e. the word embeddings of documents and averaged terms generated from the dictionary in vector space) even at instances when they do not share exact words [62]. One example for the general principle can be seen in Fig. 2 when the relative cost of moving components in tweets (*T*_1_ or *T*_2_) towards a target concept (*T*_pseudo_) is shown. As overall the cost for the first tweet is relatively lower, *T*_1_ can be taken to engage more with the concept.

The calculation with the CMD is based on the “Relaxed Word Mover’s Distance” [63] which tries to find the minimum cost to transform the embedded words of a specific document to words from other documents in the embedding space [62]. In this vein, the CMD algorithm allows us to determine the similarity of the words in a tweet document and “pseudo”-document which relates to theoretical concepts of interest and must not necessarily be of equal length. It returns a list of standardised distances which are inverted for the convenience of interpretation. With CMD, the cost (i.e. cosine similarity) of moving concepts in vector space is assigned as incoming and outgoing weights to a document (see [62]). Hence, semantically similar concepts that appear closer in vector space (i.e. they require less “effort” to be moved) can be classified, respectively, based on these weights as CT (relating to the CT dictionary) and coronavirus-specific (based on the coronavirus dictionary). One of the major advantages of this technique is using the relational word meaning for assigning common groups, instead of relying on discrete, entirely a priori determined CT categories.

More specifically, for each of the five categories in the conspiracy dictionary, a centroid (the averaged concepts) in the word embedding is calculated. Next the distance from each “tweet document” of the CT posters to the centroid is calculated with the CMD (with large CMD values indicating large concept engagement). For interpreting the results of this procedure, we adopted a threshold of ≥ 0.8 for the closeness to conspiracy categories. To ensure, for the CT posters a genuine focus on coronavirus concepts, without containing potential CT content, juxtaposed pairs for these two semantic directions are constructed [62]. For this, the semantic direction was combined with the concept mover’s distance. That is, a list of antonym pairs was generated based on the general COVID-19 dictionary and then a subsample of equal size from the CT dictionary was drawn that pose theoretical antonyms to the general COVID-19 terms. In this context general COVID-19 vectors are treated as “additions”, whereas conspiracy vectors as “subtracts”. This involves treating CT concepts (e.g. “scamdemic”, “biowarfare” or “ankle_monitors”) as antonyms to general coronavirus concepts (e.g. “vulnerable_people”, “vaccine” or “stay_home”). In a next step, the difference between the respective vectors was obtained and averaged. Eventually, we obtained one side of the continuum, which can then be quantified regarding its distance to the tweet documents with the concept mover’s distance algorithm, with a threshold of ≥ 0.8.

We validated the CMD-based classification for the CT group by randomly sampling 100 tweets (50 each for the CT-classified tweets as well as the non-CT-classified tweets). We further annotated them and compared human and CMD-based classification. This resulted in a classification by CMD with a precision of 0.8, by which 10 tweets were found, mostly due to their brief length, to be non-CT content by human annotators (i.e. false positives) and 40 were true positives. Further, this resulted in a recall of 0.89 (with 5 tweets being classified as false negatives). This eventually results in a fair *F*-measure of 0.84.

### Time series analyses

We used a variety of approaches to investigate the time course and temporal dynamics of tweets both on an aggregate level (RQ2 and RQ3) and on the individual level (RQ4). The percentage of CT-related content was calculated on a daily basis. Hence, if on a certain day a user has posted 20 tweets and half of them were CT-related, the relevant number is 0.50 for the respective day. This allows us to use the person as the reference system which enables estimating meaningful within-person trajectories (see RQ4). Hence, an increase of the involvement with CT across time would be visible in an increase in the proportion irrespective of how large the overall number of tweets was. With regard to the minimum number of tweets, this was zero due to the days on which there was no posting behaviour. The goal was to measure the CT content per user per day—not the degree of CT conviction (behind the posts) for which setting the value to zero would have been inappropriate.

First, we plotted the aggregate time series to facilitate illustration and visual exploration, thereby obtaining a first indication of linear or nonlinear trends and the occurrence of seasonal variations. Next, we calculated the autocorrelation function and partial autocorrelation function due to two substantive reasons—that is, to judge the level of inertia (positive autocorrelation) versus bouncing (negative autocorrelation) of the behaviour as well as to evaluate whether the estimated time series models require enlargement by an ARIMA (autoregressive integrated moving average) component [10]. Further, formal evaluation of seasonality was based on a decomposition of each series into trend, season, and error [11].

Second, we formally tested for linear and nonlinear trends, season effects, and potential group differences by means of generalised additive models [GAMs, 12, 64, 65]. GAMs extend generalised linear models by estimating nonlinear relationships between variables by smooth terms that can fit any degree of wiggliness. A penalty parameter prevents overfitting, with the result that the estimated curve is not wigglier than necessary. In a time series model, smooth terms can be estimated for the trend as well as nonlinear seasonality effects. A comparison with a linear trend model by means of an analysis of variance allows us to statistically differentiate both models. We estimated the GAMs with cubic regression splines and, as the dependent variables of interest were proportions (i.e. of CT-related content in the total number of tweets), we used a beta error distribution with a log link. In the case of RQ2 that involved a comparison between both groups (i.e. the non-CT group and CT group), we estimated differences in nonlinear trends and season effects by means of factor-smooth interactions.

Third, for RQ3, we conducted a structural break analysis [13] to explore potential breaks in the level or trend of a respective series. This was done to re-evaluate the causes of a nonlinear overall trend tested in the prior GAM but also to gain insights into critical events that prompted a rise in the proportion of CT-related content.

Fourth, we used a combination of time series approaches and *generalised additive mixed models* (GAMM) to analyse RQ4. Time series approaches consisted of the estimation of the degree of autocorrelation for each individual series in the CT posters, and the GAMM aimed at testing the overall nonlinear trend and seasonality as fixed effects and inter-individual differences in levels, slopes, and nonlinear functional forms by means of random effects. The differences in the level, slope, and nonlinear trends were tested following recommendations from the overall literature on multi-level models [66] and growth curve analyses [67]. Hence, we built the model in three steps.

In the first step, we tested a random intercept model incorporating a nonlinear time trend, estimated with cubic spline basis functions with *k* = 100 and a weekday predictor, estimated with thin-plate basis functions and a *k* = 7 weekdays. The number of basis functions were investigated by using the *gam.check* function in the *mgcv* package [68], which indicated that higher numbers were unnecessary. Adding a random intercept tested for significance differences in the starting point of the individuals’ timelines. The second step added a random slope. This step still contained the same nonlinear (fixed) trend for all persons but allowed for different trend strengths (i.e. slopes). Technically, the random slope was represented by a smooth interaction between the individual and the trend variable. Finally, the third step, replaced the former random effects with a random smooth component, allowing for individual differences in the functional form of the trend including intercept and slope differences. Residuals of the final model were checked for signs of autocorrelation which were not indicated.

#### R-packages

Analyses were conducted with the R version 4.0.4. Further, we used the R-package *rtweet* 0.7.0 for harvesting the Twitter data [69]; *tweetbotornot* 0.1.0 for bot classification [52]; *text2vec* 0.6 for the GloVe word embedding [57]; *CMDist* 3.0 for concept mover’s distance [62], *tidyverse* 1.3.0 for general data wrangling [70]. For the time series related analyses, we used *tsibble* 0.9.3 and lubridate 1.7.9.2 for time-based data wrangling, *mgcv* 1.8-33 and *nlme* 3.1-152 for the GAMs, *feasts* 0.1.7 for the series decomposition and feature extraction, and *strucchange* 1.5-2 for the structural break analysis.

## Results and discussion

### Description

The dataset comprised 109 individuals for each group, respectively, who had posted *N* = 595,751 status updates in total. Regarding the temporal behaviour of users, the CT group posted more tweets on average daily basis over the year than the non-CT group while showing a higher proportion of CT-related content in comparison with COVID-19-related content (see Table 2).

**Table 2:** Descriptive statistics of tweets by account for the CT group and non-CT group

Tweet characteristics per user	CT group			Non-CT group		
	*M* (SD)	Min	Max	*M* (SD)	Min	Max
Number of daily tweets	11.70 (11.60)	0.27	70.00	2.56 (1.79)	0.05	9.13
Number of daily corona-related tweets over the year	0.59 (0.69)	0.01	3.62	0.46 (0.57)	0.01	3.85
Proportion of corona-related tweets over the year	.04 (.02)	0.003	0.11	.12 (.12)	0.005	0.59
Number of conspiracy-related tweets over the year	5.33 (6.07)	0.11	44.9	–	–	–
Proportion of conspiracy-related tweets over the year	0.35 (0.13)	0.06	0.65	–	–	–

### Identifying semantically similar expressions of CTs (RQ1)

Concerning the first research question, the narrative themes contained in tweets from the CT posters were not restricted solely to motifs centring around the pandemic or lockdown measures but emerged in a variety of broader motifs (see also Samory and Mitra [4]) including: (i) events (e.g. “9/11”, the killing of George Floyd), (ii) elections (Democratic and Republican party politics), and (iii) domestic politics (“Hunter Biden scandal”), (iv) globalisation (e.g. “global communism”), (v) intelligence operations (e.g. military operations, bioweapons), (vi) media (“mainstream media”), or (vii) mystic rituals and paedophile rings (e.g. cabal, satan). Prototypical tweets for each category are provided in Supplementary Materials 2.

As aforementioned, the tweet content can be interpreted as touching the psychological needs of the person (i.e. existential, epistemic, or social needs). In this vein, when exploring the GloVe vector of the CT posters for similar vectors denoted by the cosine similarity (i.e. “*c*”), we were able to identify different realisations of these needs. Specifically, we considered cosine similarity values higher than 0.40 as indicating sufficient similarity. Further, values in the range from -1 to 0 by which values approximating 0 indicate low semantic similarity and conversely approaching 1 indicates high semantic closeness. Values with opposite polarity show diverging meaning.

For instance, threats of existential needs could be related to common uses of keywords such as “vaccinations” (*c* = 0.45 with “depopulation agenda”). These were connected with motifs referring to coalitions like pharma (*c* = 0.44 with “big_pharma”). Likewise, the keyword “deep_state” was associated with “hardware_us” (*c* = 0.40).

The notion of harm and threat was further taken up by a pervasive disapproval of media outlets, which is framed as a source of disinformation and a vehicle played by third parties to control the population (e.g. “news” was associated with “fake_news”, *c* = 0.56 or “dangerous_lunacy”, *c* = 0.43). Further, events like “9/11” (e.g. related terms were “pacification_psyop”, *c* = 0.46; or “majority_murders”, *c* = 0.44) were framed as staged and spun in a hidden fashion by “government insiders”. Other sources of threat were prominent individuals (like Bill Gates) who were depicted as being in a quest for domination and personal gain (e.g. “satanic”, *c* = 0.49; “overseas_spying”, *c* = 0.40). Emerging current social movements like the Black Lives Matter (“blm”) movement were incorporated into a threat narrative (e.g. “blm_antifa”, *c* = 0.59; “terrorists”, *c* = 0.45; “marxist”, *c* = 0.47).

Relating to epistemic needs, pragmatic markers indicate the individual attention, assessment of causes and commitment to stances [71] (e.g. “uncover” is associated with “growing_totalitarian”, *c* = 0.40 or “batresearch_program”, *c* = 0.40). Further, the element of secret agency and operations is used (e.g. “op” is associated with “chemicals_manufactured”, *c* = 0.42; “trees_changed”, *c* = 0.41; “programming_people”, *c* = 0.40). In this vein, clear goals are set, like ending child trafficking or ending the lockdown and uncovering the truth beneath the surface. Researching information is turned into a game to solve the secret plot (essentially finding proof for why the official account is not true) and in the realms of satisfying social needs by belonging to those who see through (e.g. “research” was associated with “pedo_city”, *c* = 0.48; “proven_scam”, *c* = 0.45; “public_surface”, *c* = 0.41). Henceforward, rhetorical tropes and epistemic markers of truth propositions and questioning coincidences play a functional role in engaging with conspiratorial ideation and further, as they are unlikely to be banned or shadow-banned, as a marker of shared interpretive frames in a consistent manner.

### Group comparison of posting behaviour on overall COVID-19-related tweets (RQ2)

The second research question centered around whether tweet posting behaviour focusing on general coronavirus content differed between the CT group and the non-CT group.

**Fig. 3:**
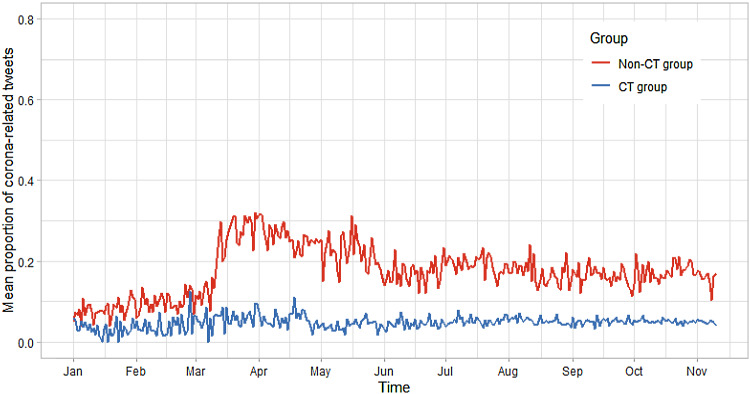
Mean proportion of COVID-19-related tweets by the CT group and the non-CT group

Figure 3 shows the proportion of coronavirus**-**related tweets to the overall number of tweets in each group. As clearly depicted in the figure, from January to March both groups tweeted to a similar extent. From March onward, the non-CT group showed a constantly higher proportion of tweets than the CT group. In addition, there was a substantial increase in the proportion of tweets at the beginning of March. Three probable events causing the increase are first, extensive media coverage of the events in the northern regions of Italy (at the time, this was the area most affected by COVID-19 besides Wuhan in the Hubei Province, China), second the announcement of a countrywide quarantine in Italy on March 9 and third subsequently the official declaration of the corona crisis as a pandemic by the WHO on March 11. Hence, particularly for the non-CT group, the “spread” of the coronavirus became prevalent in the following months (with associated terms such as “spread_covid19”, *c* = 0.66; “stop_spread”, *c* = 0.57; “prevent_spread”, *c* = 0.58). Further, the declaration as a national state of emergency in the USA resulted in public responses (for the non-CT group the GloVe model contained “emergency” associated terms like “health_emergency”, *c* = 0.54 or “state_emergency”, *c* = 0.48). As the subsequent analyses will show, the CT group in contrast focussed primarily on CT-related tweets.

With regard to temporal characteristics of the two series in Fig. 3, we estimated the autocorrelation and conducted an STL decomposition into the (nonlinear) trend, seasons, and remainder for both groups. The result was a substantially higher autocorrelation of the non-CT group (*r* = 0.83) than the CT group (*r* = 0.11), implying a stronger persistence in the posting behaviour from one day to the next. The STL decomposition suggested a weekly season effect with the tweet behaviour constantly high from Mondays to Thursdays and then rapidly dropping for the non-CT group. In contrast, no systematic pattern could be observed for the CT group. Based on the results from the estimation of the autocorrelation, we used the auto.arima function in R’s *feasts* package to identify potential ARIMA models, and a model with no autocorrelated error structure was preferred.

In the last step, we analysed differences between both groups in the dynamics of the series using a GAM with a factor smooth interaction. We used cubic splines as the family of basis functions for the time trend and thin-plate splines for the weekday. Further, we set the number of basis functions to the highest possible number that led to a converging model. This was *k* = 305 for the time trend and *k* = 7 for the weekday season (i.e. the changing pattern across the week). We estimated two models. The first was a *separate-smooth model*, which results in the estimation of a season smooth and a time trend smooth separately in each group. The second model was a *difference smooth model* which—analogously to dummy interaction—estimates baseline smooths for the season and time trend (of the non-CT group) and difference smooths for the contrasting group (i.e. the CT group). Table 3 shows the results of the separate smooth model.

**Table 3:** Results of the GAM investigating differences between the non-CT posters and CT posters in overall coronavirus-related tweets

	Separate smooth model	
	*B*	*p*
*Linear part of the model*		
Intercept	− 1.61***	< .001
Group: CT group	− 1.53***	< .001

	EDF	*p*
*Nonlinear part of the model*		
Non-CT group: season weekday	3.26***	< .001
CT group: season weekday	1.00	.750
Non-CT group: time trend	17.91***	< .001
CT group: time trend	46.34***	< .001
*R* square	.92	
Deviance explained	.95	

EDF = Effective degrees of freedom (indicates the amount of wiggliness of a curve); EDF = 1 indicates a straight line; ***p < .001

Table 3 displays two types of coefficients. The coefficients in the linear segment are the regression intercept and the level difference between the CT group and the non-CT group. The EDF (effective degrees of freedom) in the nonlinear segment describes the nonlinear dynamics in the weekday effect and the overall time trend. The table shows that the non-CT group showed a significant weekday effect, while the CT posters did not. In addition, both groups showed a nonlinear trend which was significantly wigglier for the CT group (*p* < 0.001).

### Identifying dynamics of CT-related tweets (RQ3)

**Fig. 4:**
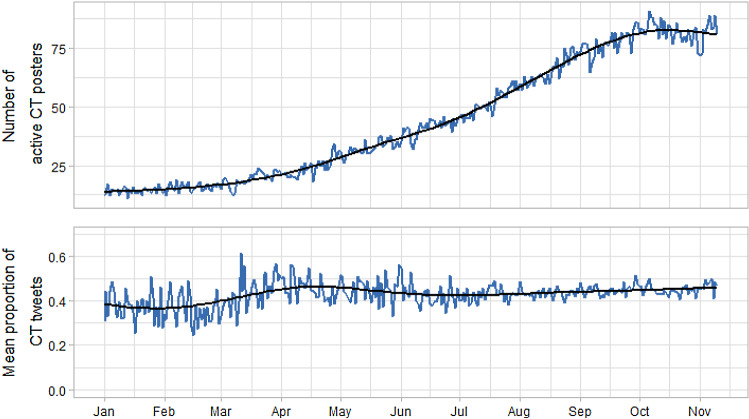
Distribution of CT posters (upper panel) and mean proportion of CT tweets (bottom panel)

The third research question focussed on the time series of CT-related tweets and its characteristics. Figure 4 shows the distribution of the number of CT posters across time (upper panel) and the proportion of tweets (lower panel) of this group. The figure shows that that number of individuals increased during 2020, revealing a horizontal spread of CT engagement (i.e. the number of involved persons), whereas the proportion showed an increase in the spring and a seemingly constant level for the rest of the year (we will re-consider this later regarding structural breaks). A preliminary GAM, not yet considering potential autocorrelation and seasonality, revealed a significant positive linear trend for the number of users (*B* = 0.006, *p* < 0.001) and proportions of tweets (*B* = 0.0009, *p* < 0.001) signifying an 8% increase from January to November. Furthermore, specifying a nonlinear trend in three GAMs resulted in a significantly better data fit than the linear variants in all three cases. It should be noted, however, that the number of users being represented on each day differed across the time, which cannot be reflected in the single-series GAM. This will be considered in the section on individual trends and their averages.

The next two steps focussed on autocorrelation and seasonality. The autocorrelation function revealed a mean lag-1 autocorrelation of *r* = 0.34 for the tweets. Although this correlation was higher compared to the tweets with general COVID-19-related content (*r* = 0.11), this still indicated a lack of substantial autocorrelation (especially if compared to the *r* = 0.83 in the non-CT group). When estimating the GAM, we found that a simple model without an autoregressive and moving average component would best fit the data—therefore, we repeated the GAM by only including a weekly seasonal smooth that had been suggested by the STL decomposition. The results showed, however, no significant seasonality. Hence, the interpretation of the aforementioned seasonal pattern should be undertaken with caution. Overall, these analyses suggest that the posting behaviour of CT-related content contains a high degree of randomness and day-specific dynamics.

**Fig. 5:**
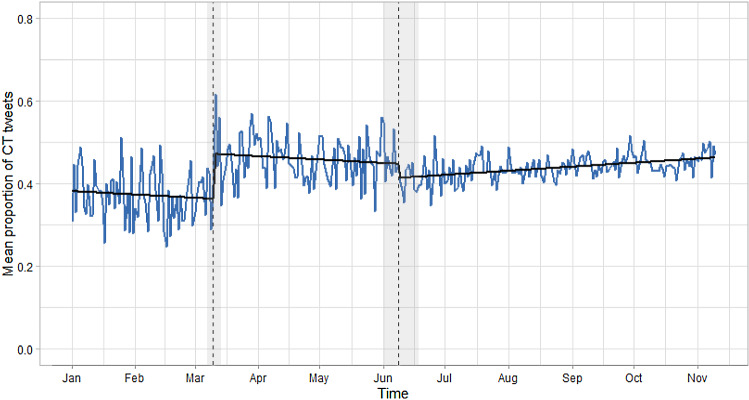
Structural break analysis of CT tweets for the CT group via the CUSUM and F-test. Grey areas indicate confidence intervals for two structural breaks on 10 March 2020 and 8 June 2020 (dashed lines)

As the final analysis, we conducted a structural break analysis by investigating structural breaks in linear trends within segments. Despite the non-significant season effects, we based this analysis on the residuals of a former GAM with a nonlinear season estimate but no trend. A test focusing on the cumulated sum of standardised residuals (CUSUM fluctuation test) and the *F*-test (*F* = 77.11, *p* < 0.001) indicated a significant deviation from the null hypothesis that all measures are reflections of the same data generating process. A subsequent analysis of variants with differing numbers of breakpoints showed that the Bayesian information criterion (BIC) suggested two breakpoints, whereas the residual sum of squares indicated that all models with more than one breakpoints had equal fit. Figure 5 shows the breakpoints and their confidence intervals (i.e. the grey area) for CT tweets of the CT group.

The dates associated with the breakpoints were March 10 and June 8. Noteworthy events in this time span are on the one hand the implementation of public health measures on 8 March 2020 which resulted in strict social distancing measures in highly affected European countries like France or Spain. This was followed by the WHO pandemic declaration. The second breakpoint falls into the time of the emerging “Black Lives Matter” Movement on June 3 and the George Floyd protests and tearing down of memorials in several other countries during the following days.

Furthermore, the division of the series in three segments resulted in non-significant trends in the first two segments (both Bs = 0.0002, *p* = 0.46 and *p* = 0.24, respectively) but a significant trend in the phase beginning on June 9 (*B* = 0.0003, *p* < 0.001). This effect, however, should not be overinterpreted due to the substantially higher power compared to the trend estimates in the first two segments.

### Individual differences in temporal characteristics (RQ4)

In addition to the analyses of the tweets on an aggregate level, we investigated the series of individual CT posters. As a consequence of the Twitter API regulations on the amount of available historical tweets of individual timelines, we had series that varied in length, with an average length of 174 days (SD = 92), ranging from 37 to 315 days. Whereas the former analyses presented information about the overall dynamics of the aggregate posting behaviour, the following analysis focussed on inter-individual differences in the dynamics, including differences in the autocorrelation, level, slope, and functional linear and nonlinear trends.

With regard to inter-individual variations in the autocorrelation, we found substantial differences ranging from − 0.34 to 0.55 (*M* = 0.10, SD = 0.15). The first pattern was most often a result of switching between days on which a person tweeted CT content followed by one or several days of either not tweeting at all or tweeting only non-CT content. The positive autocorrelation consisted of consecutive series of days on which the person posted followed by several days of absence.

As Table 4 reveals, the Akaike information criterion (AIC) was lowest for the random smooth model, indicating significant differences in the nonlinear dynamics between individuals. Furthermore, the explained variance was low for all models showing the large individual deviations, often spanning the range between zero tweets per day (and accordingly zero proportion of conspiracy content) up to 100 percent CT content. Finally, while the fixed effects for the time trend revealed a nonlinear average trajectory across time, there was no significant weekday effect.

**Table 4:** Results of the generalised additive mixed-effects model addressing inter-individual differences in time trends

	Random intercept model		Random slope model		Random smooth model	
	EDF	*p*	EDF	*p*	EDF	*p*
*Fixed effects*						
Time trend	7.77***	< .001	7.77***	< .001	7.50***	< .001
Weekday	2.36	.083	2.34	.092	2.34	.095
*Random effects*						
Random intercept	102.80***	< .001	77.85***	< .001		
Random slope			71.42***	< .001		
Random smooth					266.14***	< .001
R square	.139		.156		.180	
Deviance explained	.009		.009		.010	
AIC	− 423,743.1		− 424,046.6		− 424,468.2	

EDF = Effective degrees of freedom (indicates the amount of wiggliness of a curve); EDF = 1 indicates a straight line; ***p < .001; AIC = Akaike information criterion

**Fig. 6:**
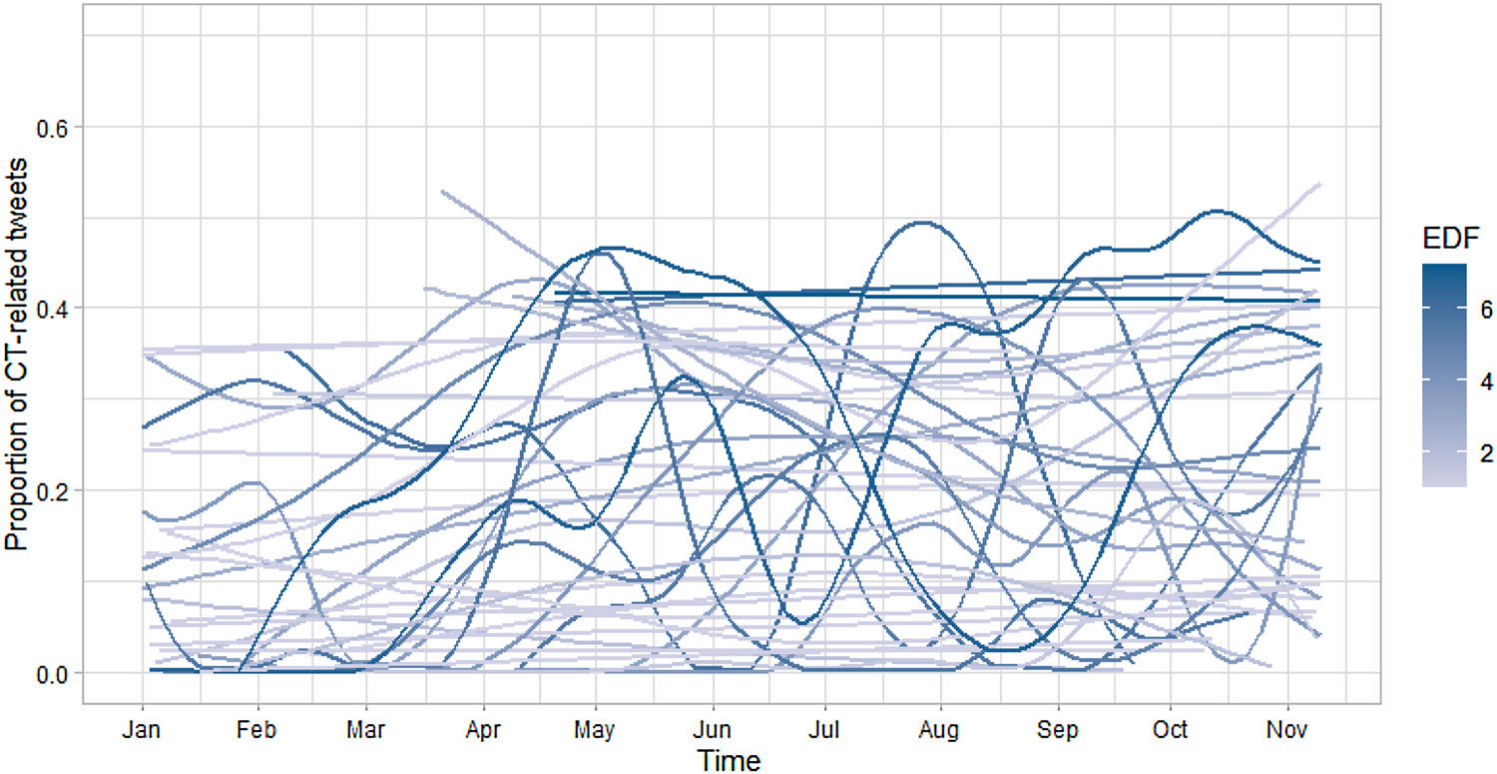
Proportion of CT-related tweets and effective degrees of freedom (EDF) for a subsample of individual CT posters (at least 200 days of posting behaviour)

To analyse the individual nonlinear trends and to judge the percentages of individual positive versus negative linear trends, we estimated specific single-person GAMs for the CT posters. To apply a comparison standard and not to overwhelm the depiction, Fig. 6 shows the trends for those individuals for which at least 200 days of data were present. The figure shows the immense differences in level and nonlinear trends across time.

To draw a conclusion about which percentage of the CT posters systematically increased or decreased the proportion of CT content, we estimated a GAM with a linear time trend while controlling for a nonlinear weekday effect. The regression coefficients had a mean of *B* =  − 0.001 (SD = 0.012) with a min of *B* =  − 0.06 and a max of *B* = 0.04, which showed no overall trend but also inter-individual differences in the increase versus decrease of posted CT content.

## Discussion

In the present article, we investigated the spread of CT tweets on Twitter throughout the first year of the COVID-19 pandemic in 2020. We used state-of-the-art text analytics (word embeddings) and time series analyses on Twitter timelines of 109 CT posters and non-CT posters, respectively, to investigate the content of CTs as well temporal characteristics of aggregate and individual series. Results showed that CT tweets fit well with claims of scholars emphasising the role of violation of existential needs in endorsing CTs [9, 72]. In this regard, CTs can be interpreted as the individual’s attempts to cope with an uncertain and dangerous situation and to attribute causes to external agents in order to gain control. While CTs have been shown to involve cognitive biases, they can be seen as evolved patterns to cope with existential threats and perceived powerlessness. This prepares the ground for user-generated content that refers to considerably few lucid coalitions (e.g. abstract references to the government, media, or concrete ones like Anthony Fauci) that are adapted to new events occurring.

### The use of word embeddings to identify CTs and broaden conceptual knowledge

Our approach of using word embeddings, informed by a minimal set of theoretical constructs (agency, pattern, coalition, secrecy, threat), resulted in the identification of terms with related semantic meaning that further enrich our knowledge on conspiratorial worldviews and implicit language use. In finding CTs in which either the severity or existence of the pandemic is called into question (i.e. hoax) or that blame certain actors for causing the pandemic (i.e. Bill Gates, China, deep state), as a way of a collective sensemaking of events, our results align with those of van Mulukom et al. [9]. The latter of the two schemes exemplifies an integration with other pre-existing, conceptually unrelated CTs, for instance, relating to the “pizzagate conspiracy”, anti-vaccination, “9/11 inside-job” or QAnon (see also [73]). These strategies might eventually steer different prevention behaviours of the posters—that is—rejecting prevention measures altogether or only partially. In a similar vein as Samory and Mitra [74] noted, albeit coalitions are easily discernible the other theoretical constructs (e.g. threat or pattern) are much more finely distinguished.

### Analysing the temporal dynamics of CT tweets

Beyond the semantic analyses, the temporal analyses resulted in insights into the temporal dynamics of CT tweets on the average level and the individual level as well as differences between CT posters and non-CT posters and within the group of CT posters. First, we found substantial differences between non-CT posters and CT posters in the series of tweets focusing on motifs centring on coronavirus-related content. In particular, the series of these tweets of the CT posters had a remarkably lower level indicating that although having the same reactivity to coronavirus-related events (e.g. rising infection rates, governmental measures), CT posters tend to strongly respond with CT-related content. Hence, both groups differ on the abstraction level of their responses. This is most apparent when integrating the results of Figs. 3 and 4 to show that the CT group posted fewer tweets containing non-CT content compared to the non-CT posters.

As a second substantive result, the time series indicated a strong dynamic in the posting pattern of users in the CT group indicating a substantial impulsiveness of the posting behaviour. This was evidenced by a significantly stronger wiggliness of the overall series, the much lower autocorrelation, the lower level of weekly seasonality, and the lack of residual autocorrelation. The latter suggests that the behaviour of CT posters is an impulsive reaction to day-level events and not a step-wise and sustained distribution of CT content. This aspect has implications for the evaluation of the role of Twitter as a facilitator of an individual self-radicalisation. The latter is also indicated by the negligible trend in the proportion of CT-related tweets across 2020. This result shows that the posting behaviour of the CT group as a collective does not indicate a disequilibrium or imbalance but can rather be represented as a stochastic process.

While these results concern aggregate level of analysis, analysing the individual level revealed a more complex and diverse picture. The analyses revealed substantial differences in the level of inertia—indicated by the strong differences in the individual autocorrelations—as well as the trends in terms of slope and functional form. In this regard, some individuals showed a linear upward trend and others a strong, dynamic reactivity. However, for those exhibiting a linear upward trend, this trend again was not substantial. The wiggliness of some series suggests that these individuals were more reactive to daily stimulations. In line with existing theory, this finding can be explained by the internally driven pattern of behaviour shown by a CT-prone person. This type of person is trying to make sense of the news he/she receives with the ultimate goal to fulfil his/her epistemic, existential and social needs by showing hyperactive pattern recognition, which turns into the maladaptive behaviour of endorsing conspiracy beliefs [1]. This erratic hyperactivity is striking if compared with the aggregate trend of the non-CT posters. This group showed greater inertia and more consistent engagement with mainstream content at the aggregate level, a behaviour pattern in stark contrast to the erratic reactivity of the CT posters.

### Future research implications

Beyond providing the insights discussed before, our results may stimulate future research that addresses issues that are beyond the scope and possibilities of the present paper. First, as discussed above, our results point to a posting behaviour that can best be described as a stationary stochastic process, which again may be interpreted as a signal of calm in the ongoing discussions about social media, the spread of conspiracy convictions, and false narratives. It should be noted, however, that this interpretation only concerns the number of users sampled within this study and their behaviour but not a potential spread of conspiracy information and growth of social networks across future CT users. In this regard, one result in this study was the strong upwards trend of the number of users that—implied that the bulk of users sampled had emerged in the later part of 2020. Hence, we recommend investigating the potential divergence between personal radicalisation processes and a nonetheless possible spread of CT content.

Second, the remarkable inter-individual differences point to individual or contextual determinants of these differences. In this regard, our study lacked the data to further investigate such determinants, most probably by integrating social media data with individual-level data from, for instance, surveys. Survey data have a long tradition in the social sciences and allow researchers to measure relevant constructs (e.g. personality traits, political attitudes, demographic information) in a reliable and valid manner. Methodologically, such questions can both be approached by using modern multilevel models (e.g. with such person factors predicting features of the individual series of tweets (e.g. trend, wiggliness, inertia) as well as typological or cluster-based approaches targeting the identification of groups of individuals with a similar radicalisation process. Furthermore, the validity and robustness of discriminating conspiracy content from non-conspiracy content should be further corroborated by comparing our results to baselines.

### Limitations of the study

The present study is confronted by some limitations which, although not critical for the main results of the study, should be taken into consideration for future research. First, Twitter’s API rate limit led to timelines that differed substantially in the time span of retrieved content. As a consequence, an individual’s time span showed a moderate albeit significant correlation (*r* = − 0.25, *p* < 0.001), indicating that longer series were wigglier than in cases where the contingent of tweets were spread more evenly across the time span. Without representing a limitation per se, we note that one of the influences on the series’ dynamics may not be psychological but technically based.

Second, our results may be specific to the COVID-19 pandemic and not generalisable to other forms of societal events and their interpretation. Likewise, we recommend a careful interpretation of our study for CT processes beyond posting on Twitter, as these users may represent a sample that is not representative of the general population [75, 76]. Hence, future research should investigate longitudinal processes of other platforms for measuring CTs as well as the key differences between the Twitter population and other populations. Likewise, extreme CT-prone persons may be banned from Twitter or adapt their behaviour so as not avoid being banned—thus, indicating an example of proxy population bias [75].

Third, another restriction on sample representativeness is implications of tweet deletion and account suspensions. A potential result of users deleting their tweets or accounts, setting accounts to private, or becoming suspended due to a violation of Twitter’s Terms of Service might lead to the underrepresentation of misinformation content in a data sample (see [77]). Thus, it needs to be acknowledged that the true rate of conspiracy content might be higher than stated and users with high trends in their postings might be missing. One step towards ameliorating this problem is concentrating further on sampling user timelines with the REST API and adding a real-time component by refreshing the dataset over time and comparing changes which are due to deletion for this time period. This could be a viable approach for conducting historical tweet analyses, given that anonymity and ethical principles for users are considered [77]. Finally, a strength of our study is that it is founded on scholarly definitions of CT properties that are general and scalable and, hence, offers several implications for further research. Specifically, our analysis pipeline proved to be suitable for matching theoretical expectations in terms of both user’s group and individual behaviours. Future research could take up, on the one hand, on contextualised word piece embeddings that mitigate issues of word sense disambiguation and provide bidirectional contexts (e.g. with Bidirectional Encoder Representations from Transformers) and, on the other hand, temporal word embeddings that allow for modelling language evolution, for instance, with probabilistic state space models where the word and context embeddings evolve with time. Such outlooks on word evolution may provide information to perspectives of how users adapt language as an indicator of increasing radicalisation. Such an approach could provide further information on the change of content meaning over time and provide fine-grained insights into emotional responses which evoke responses at short time intervals (e.g. minute scale). This type of semantic and temporal approach can add valuable information to theoretical assumptions on feelings of anxiety and lack of control, which have been the focus of survey studies [8] to date. Taken further, when considering temporal dependencies of emotions on social media, dynamic modelling techniques for studying within-person processes can be fruitful. Eventually, using a case–control design holds the potential of inquiring causal questions, as to comparing the impact of certain events, user characteristics or social factors on user behaviours.

## Supplementary Information

Below is the link to the electronic supplementary material.

Supplementary file 1 (DOCX 2321 kb)

## Data Availability

Data are available upon request.

## Code availability

Code is available upon request.
