# Supplementary material for: Conspiracy theories on Twitter: emerging motifs and temporal dynamics during the COVID-19 pandemic
Source: Int J Data Sci Anal. 2021 Dec 24;13(4):315–33. doi: 10.1007/s41060-021-00298-6 (PMC8703214; doi:10.1007/s41060-021-00298-6)
Supplement: Supplementary file 1 — (DOCX 2321 kb) [file 41060_2021_298_MOESM1_ESM.docx]

**International Journal of Data Science and Analytics**

**Conspiracy theories on Twitter: Emerging motifs and temporal dynamics during the COVID-19 pandemic**

Veronika Batzdorfer¹, Holger Steinmetz¹, Marco Biella², Meysam Alizadeh³

**Supplementary material**

¹ Big Data Department, Leibniz Institute for Psychology (ZPID), Trier, Germany

² Department of Psychology, Eberhard Karls Universität Tuebingen, Tuebingen, Germany

³ Kennedy School of Government, Harvard University, Cambridge, USA

Corresponding author

Veronika Batzdorfer, Email: vb@leibniz-psychology.org

# Twitter sampling

## Constructing keyword queries

**Table 1**

*Literature resources for COVID-19 related narratives for constructing Twitter keywords*

| Keywords | Source |
| --- | --- |
| Typology of COVID-19 misinformation | Brennen, J.S., Simon, F., Howard, P.N., Nielsen, R.K. (April, 2020). Types, sources, and claims of COVID-19 misinformation. *Reuters Institute*, *7*, 3-1. <http://www.primaonline.it/wp-content/uploads/2020/04/COVID-19_reuters.pdf> |
| Wuhan lab  Bill Gates, 5G | Jiang, J., Chen, E., Yan, S., Lerman, K., & Ferrara, E. (2020). Political polarization drives online conversations about COVID‐19 in the United States. Human Behavior and Emerging Technologies, *2*(3), 200-211. <https://onlinelibrary.wiley.com/doi/pdfdirect/10.1002/hbe2.202> |
|  | Shahsavari, S., Holur, P., Tangherlini, T. R., & Roychowdhury, V. (2020). Conspiracy in the time of corona: Automatic detection of covid-19 conspiracy theories in social media and the news. *arXiv preprint* arXiv:2004.13783.<https://arxiv.org/pdf/2004.13783.pdf> |
| 5G network, secretive elites, Bill Gates, China, Russia | EUvsDISINFO. (2020). ‘EEAS Special Report Update: Short Assessment of Narratives and Disinformation around the COVID-19 Pandemic (UPDATE 23 APRIL – 18 MAY)’. EUvsDISINFO. [Accessed Nov. 2020]. <https://euvsdisinfo.eu/eeas-special-report-update-short-assessment-of-narratives-and-disinformation-around-the-covid19-pandemic-updated-23-april-18-may/> |
| Deep state | EUvsDISINFO [accessed Nov. 2020] <https://euvsdisinfo.eu/report/covid-19-is-a-biological-weapon-of-artificial-origin-created-by-bill-gates-george-soros-and-the-deep-state>  EUvsDISINFO [accessed Nov. 2020] <https://euvsdisinfo.eu/report/western-elites-implementing-their-plan-x-through-pandemic> |
| Wuhan-lab relation | EUvsDISINFO [accessed Nov. 2020] <https://euvsdisinfo.eu/report/it-is-not-plausible-that-the-covid-19-developed-naturally/> |
| New World order | EUvsDISINFO [accessed Nov. 2020] <https://euvsdisinfo.eu/report/vaccines-dont-heal-their-production-is-part-of-the-agenda-for-a-new-world-order/> |
| Bill Gates | EUvsDISINFO [accessed Nov. 2020] <https://euvsdisinfo.eu/report/microsofts-patent-666-involves-microchipping-people-in-order-to-monitor-their-daily-activity-in-exchange-for-cryptocurrency/> |
| Globalist elites | EUvsDISINFO [accessed Nov. 2020] <https://euvsdisinfo.eu/report/the-global-elites-want-to-reduce-the-excessive-numbers-of-useless-eaters/>  EUvsDISINFO [accessed Nov. 2020] <https://euvsdisinfo.eu/report/western-governments-are-deliberately-exaggerating-the-coronavirus-pandemic-to-strengthen-global-corporate-capitalism-and-to-accelerate-globalisation> |

## Keyword queries for CT accounts

For each of a total of 6 keywords, a 7-month time period was sampled. That is, for each month within a keyword query a random sample of 10 Twitter users was manually identified. The respective tweet matching the keyword query along the Twitter-handle was retrieved.

**Fig. 1** Twitter web search for identifying potential CT accounts

*Note*. No keyword-based/hashtag-based searches were conducted, as only the most recent 6-9 days could be accessed with the free search query). Hence a manual Twitter Web Search for retrospectively identifying accounts was conducted. Since the Twitter search matches keywords contained as substrings the search is case insensitive. Further, in the search an English language focus is specified (lang:en).


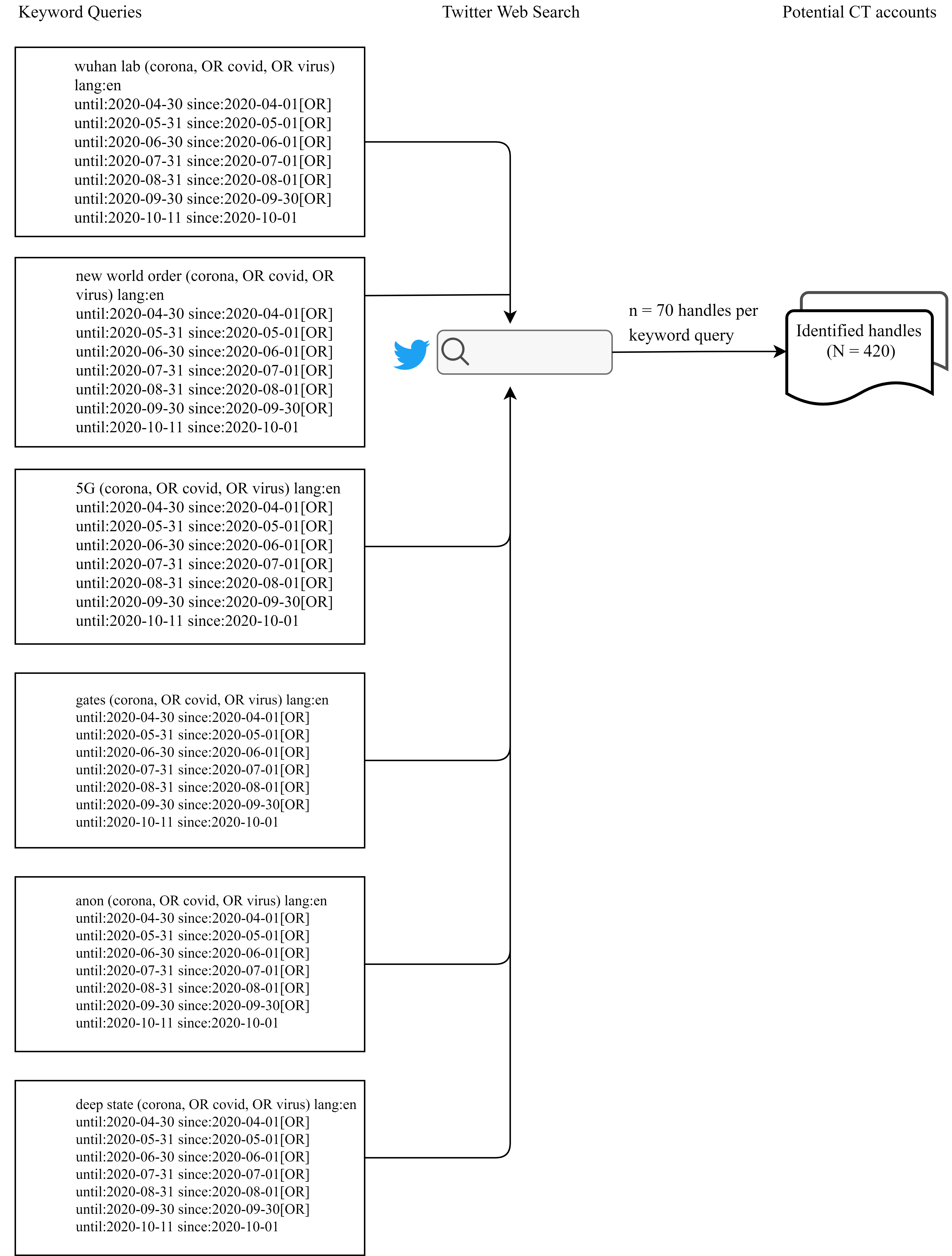


## Coding for CT account inclusion

**Table 2**

*Description of coding regime for tweets to detect potential conspiracy accounts on Twitter* (see [[1]](https://www.zotero.org/google-docs/?mWAphJ))

| ID | Variable | Explanation | Categories |
| --- | --- | --- | --- |
| 1 | agency | actions underlie malicious motives and schemes | present (=1), absent (=0) |
| 2 | coalition | a hostile group (majority (e.g. governmental institutions, nations) or minority (e.g. ethnicities), or powerful individuals operate | present (=1), absent (=0) |
| 3 | pattern | a causal relation about entities exists (e.g., paranormal beliefs) | present (=1), absent (=0) |
| 4 | secrecy | agents act covertly, cannot be validated | present (=1), absent (=0) |
| 5 | threat | actors are potentially harmful to individual/ group | present (=1), absent (=0) |

*Note*. Two coders rated each tweet whether variables (1-5) were present or absent. Based on at least three variables present, an overall rating for inclusion or exclusion was made. In case of disagreement resolution was sought based on the variable rating and the individual user profile information or the historical timeline was consulted.

**Table 3**

*Example coding of tweets for inclusion of CT accounts*

| Keyword | Text | 1 | 2 | 3 | 4 | 5 | I/E |
| --- | --- | --- | --- | --- | --- | --- | --- |
| 5G | They have scared everyone into their homes so they can install the kill grid all around us. People don't just fall over and die from a virus like they were in doing in China. But they do if their body cannot absorb oxygen. 5G starves the body of oxygen. Face with symbols over mouthTHIS IS A WEAPON!Pouting face | 1 | 1 | 1 | 1 | 1 | 1 |
| deep state | These clowns don’t even know what a virus is let alone how to test for a scamdemic one. Pure Satanic violation of Natural Law occurring, and the Waitriots cheer it on! #Qanon #FauciFraud #BillGatesBioTerrorist #DeepState #Covide1984 | 0 | 1 | 1 | 0 | 1 | 1 |
| gates | BILL GATES AND GEORGE SOROS ARE TAKING OVER THE WHITES STERILISATION PROGRAMME FROM THE CLINTON FOUNDATION UNDER THE GUISE OF GLOBALIST CHINAS' COVID 19 EUTHANASIA PROGRAMME!! NO SUCH THING AS AN ACCIDENT OR COINCIDENCE!!! | 1 | 1 | 1 | 1 | 1 | 1 |
| new world order | Demonic Pedophile New World Order Puppet wants you to enslave yourself with Big Brother tracking app. Say NO to Covid 1984 | 1 | 1 | 1 | 1 | 1 | 1 |

*Note*. Example coding by one coder regarding the presence (0,1) of *agency*, *coalition,* *pattern*, *secrecy*, *threat* (1-5) in a tweet. Keyword referring to the main keyword in the Twitter query for an example month. I/E = Inclusion (1) or Exclusion (0) of respective tweet and account.

## Coding for non-CT account inclusion

**Table 4**

*Example coding of tweets for inclusion of non-CT accounts*

| Text | 1 | 2 | 3 | 4 | 5 | I/E |
| --- | --- | --- | --- | --- | --- | --- |
| As lawsuits against Purdue Pharma piled up, the worldâ€™s top universities accepted at least $60 million in gifts from the Sackler family some as recently as 2018. | 1 | 1 | 1 | 0 | 0 | 0 |
| BioNTech Chief Commercial Officer, Sean Marett, says Canada could receive Pfizer-BioNTech vaccine within 24 hours of Health Canada approval #RBL | 0 | 1 | 0 | 0 | 0 | 1 |
| Will we finally learn this lesson, asks @matthewherper: No single strategy or technology is going to rescue us from the pandemic: not masks, not better testing, not a drug, not vaccines. Fighting #Covid19 requires doing many things well. https://t.co/cujsNNaZCb via @statnews | 0 | 0 | 1 | 0 | 0 | 1 |
| @susie_parker I think this info was released by the govt? | 1 | 1 | 0 | 0 | 0 | 1 |
| Here are #COVID19 case counts for today for the counties surrounding #ShelbyCountyTN / #MemphisTN metro area. Information about other counties' case counts comes from  @TNDeptofHealth @ADHPIO and @msdh | 0 | 0 | 0 | 0 | 0 | 1 |

*Note*. Example coding by one coder regarding the presence (0,1) of *agency*, *coalition,* *pattern*, *secrecy*, *threat* (1-5) in a tweet. I/E = Inclusion (1) or Exclusion (0) of respective tweet and account.

## 1.5 Sampling flow of Twitter CT accounts


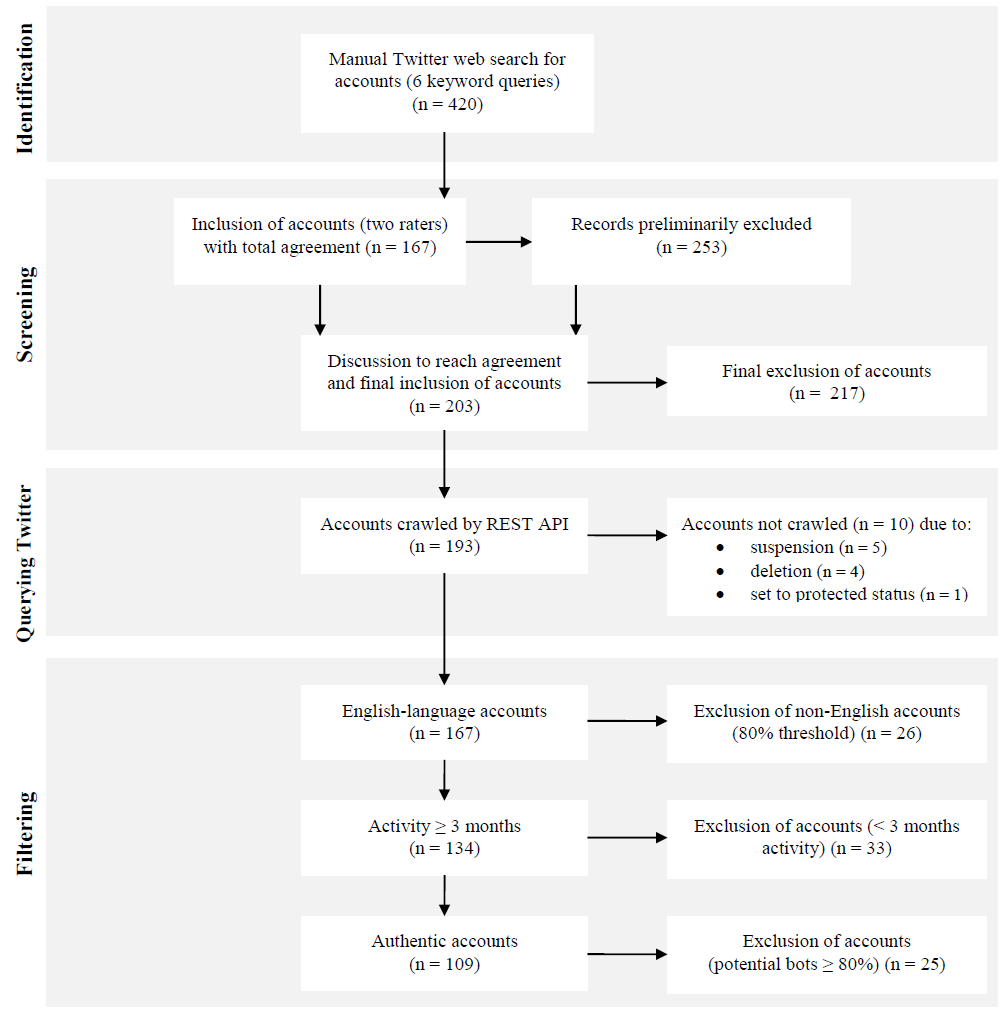


**Fig. 2** Twitter sampling flow for CT accounts

## 1.6 Sampling flow of Twitter Non-CT accounts

**Fig. 3** Twitter sampling flow for non-CT accounts


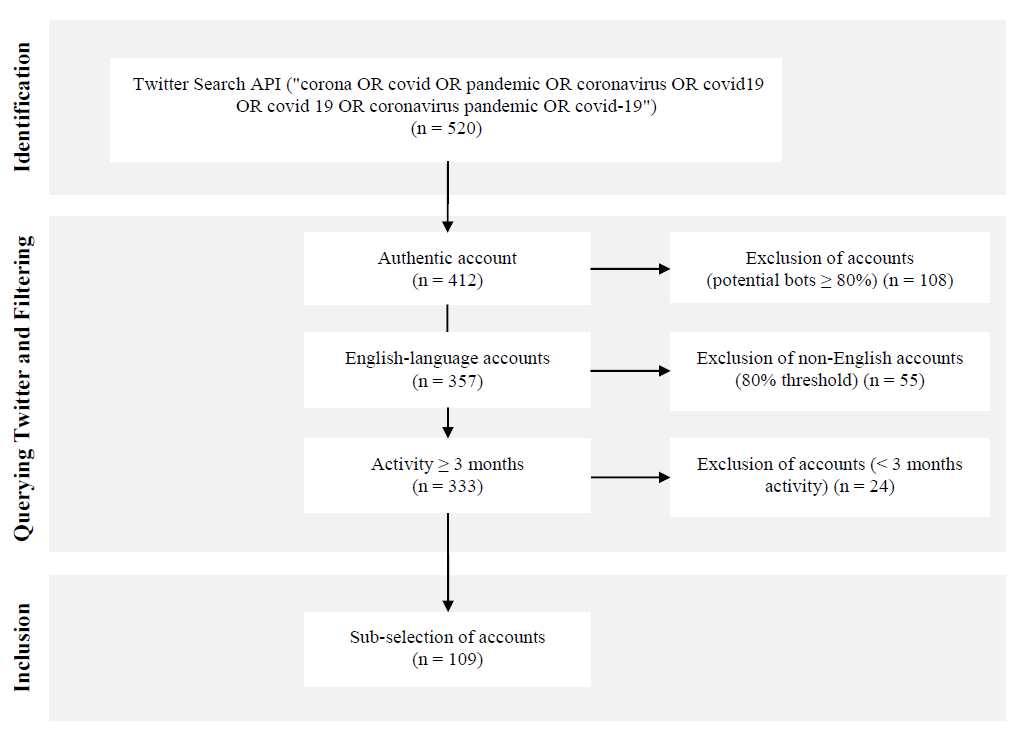


# Prototypical conspiracy tweets

**Table 5** Prototypical conspiracy patterns of tweets (adapted from [1])

| Motifs | CT Patterns | | | | | Tweet Prototype |
| --- | --- | --- | --- | --- | --- | --- |
|  | Agency | Threat | Pattern | Secrecy | Coalition |  |
| (Domestic) politics | polluted the minds, owned-and-controlled | carnage in Palestine | propaganda for decades | Why do US citizens blindly accept and endorse | AIPAC, Zionist, mainstream media | Question: Why do US citizens blindly accept and endorse the Zionist carnage in Palestine without question? Answer: The AIPAC-owned US government and the Zionist owned-and-controlled mainstream media have polluted the minds of Americans with propaganda for decades. |
| Elections | have been for eons, every lie required to build Tower of Babel | fraud | soon entire thing will crumble in on itself, follow the Bible | every lie, woke up, wake up | - | Look how many people 9/11 woke up/how many people did COVID-19 wake up?/people seeing our elections are fraud (have been for eons)..every lie required to build Tower of Babel weakens it's foundation..soon entire thing will crumble in on itself..follow the Bible and you'll be fine |
| Events | planned and executed | - | planned and executed the events of 9/11. There were no Muslims involved. | You have been lied to big time;  Research it...wake up? | Israeli Zionist,  world governments and the mainstream media | Incredibly evil Israeli Zionist subhumans planned and executed the events of 9/11. You have been lied to big time by world governments and the mainstream media. Research it...wake up? |
| Globalization | controlled by profiteering capitalists | World War,  A fight, global communism | fake communism, The Soviet Union and Red China were financed by Western free market capitalists | fake communism | The Soviet Union, Red China, Western free market capitalists | It's a World War... A fight for International Law vs. global rules-based order... aka global communism; But it is fake communism, controlled by profiteering capitalists. The Soviet Union and Red China were financed by Western free market capitalists. |
|  |  |  |  |  |  |  |
|  |  |  |  |  |  |  |
| Motifs | CT Patterns | | | | | Tweet Prototype |
|  | Agency | Threat | Pattern | Secrecy | Coalition |  |
| Intelligence | they're stridently hyping the threat of COVID19, ushering in | brainwash the masses, big psy-op | a big psy-op for ushering in new ways to control the gullible masses | psy-op | mainstream media,  Wall Street, Government | The mainstream media speaks with one voice to brainwash the masses, they're stridently hyping the threat of COVID19. The media hyperbole plus Wall Street over-reaction plus Government over-reaction looks like a big psy-op for ushering in new ways to control the gullible masses. |
| Mystics, media and government | engineer | Economic collapse and societal breakdown | Cabal script,  for enhanced control | - | media controlled outlets, Government puppet leaders | Sense the Cabal's Script to their media controlled outlets and to our Government puppet leaders: engineer an economic collapse and societal breakdown..for enhanced control #NWO |
| Corona and Bill Gates | GLOBAL DIGITAL IDENTITY system | a;BARCODE; that can be scanned anytime by the government | His system uses a special ink that's embedded in the skin during VACCINATIONS | Getting the picture? | BILL GATES | Getting the picture?: BILL GATES (CoronaVirus) funded technology for a GLOBAL DIGITAL IDENTITY system.. His system uses a special ink that's embedded in the skin during VACCINATIONS! That way YOU'LL have a;BARCODE; that can be scanned anytime by the government! |
| Corona and depopulation | implement their agenda | depopulation totalitarian government | Police forces across the world are given special powers to impose lockdowns with violent enforcement | Using […] as an opportunity | The wealthy international ruling class | The wealthy international ruling class is using the COVID19 pandemic as an opportunity to implement their agenda of depopluation totalitarian government. Police forces across the world are given special powers to impose lockdowns with violent enforcement. |

**References**

1. van Prooijen, J.-W., Van Vugt, M.: Conspiracy theories: Evolved functions and psychological mechanisms. Perspect. Psychol. Sci. 13, 770–788 (2018)
